# Supplementary material for: Overlapping cell population expression profiling and regulatory inference in C. elegans
Source: BMC Genomics. 2016 Feb 29;17:159. doi: 10.1186/s12864-016-2482-z (PMC4772325; doi:10.1186/s12864-016-2482-z)
Supplement: Additional file 13: — Web supplement. (DOC 21 kb) [file 12864_2016_2482_MOESM13_ESM.zip › sortWeb/clusters/hier.300.clusters/4.html]

Cluster 4 

## Cluster 4

### Expression

| cnd-1 rep. 1 | cnd-1 rep. 2 | cnd-1 rep. 3 | pha-4 rep. 1 | pha-4 rep. 2 | pha-4 rep. 3 | ceh-27 | ceh-36 | ceh-6 | F21D5.9 | mir-57 | mls-2 | pal-1 | pros-1 | ttx-3 | unc-130 | hlh-16 | irx-1 | ceh-6 (+) hlh-16 (+) | ceh-6 (+) hlh-16 (-) | ceh-6 (-) hlh-16 (+) | cnd-1 singlets | pha-4 singlets | 0 | 60 | 120 | 150 | 180 | 240 | 330 | 390 | 420 | 480 | 540 | 570 | 600 | 630 | 660 | NAME | Functional description |
| --- | --- | --- | --- | --- | --- | --- | --- | --- | --- | --- | --- | --- | --- | --- | --- | --- | --- | --- | --- | --- | --- | --- | --- | --- | --- | --- | --- | --- | --- | --- | --- | --- | --- | --- | --- | --- | --- | --- | --- |
|  |  |  |  |  |  |  |  |  |  |  |  |  |  |  |  |  |  |  |  |  |  |  |  |  |  |  |  |  |  |  |  |  |  |  |  |  |  | T04A6.2 |  |
|  |  |  |  |  |  |  |  |  |  |  |  |  |  |  |  |  |  |  |  |  |  |  |  |  |  |  |  |  |  |  |  |  |  |  |  |  |  | F17C11.20 |  |
|  |  |  |  |  |  |  |  |  |  |  |  |  |  |  |  |  |  |  |  |  |  |  |  |  |  |  |  |  |  |  |  |  |  |  |  |  |  | Y54F10BM.3 |  |
|  |  |  |  |  |  |  |  |  |  |  |  |  |  |  |  |  |  |  |  |  |  |  |  |  |  |  |  |  |  |  |  |  |  |  |  |  |  | K05F1.1 |  |
|  |  |  |  |  |  |  |  |  |  |  |  |  |  |  |  |  |  |  |  |  |  |  |  |  |  |  |  |  |  |  |  |  |  |  |  |  |  | T25B6.t2 |  |
|  |  |  |  |  |  |  |  |  |  |  |  |  |  |  |  |  |  |  |  |  |  |  |  |  |  |  |  |  |  |  |  |  |  |  |  |  |  | *ocam-1* | ONECUT (homeobox) Associated Motif containing |
|  |  |  |  |  |  |  |  |  |  |  |  |  |  |  |  |  |  |  |  |  |  |  |  |  |  |  |  |  |  |  |  |  |  |  |  |  |  | *linc-70* | Long Intervening Non-Coding RNA |
|  |  |  |  |  |  |  |  |  |  |  |  |  |  |  |  |  |  |  |  |  |  |  |  |  |  |  |  |  |  |  |  |  |  |  |  |  |  | Y113G7A.17 |  |
|  |  |  |  |  |  |  |  |  |  |  |  |  |  |  |  |  |  |  |  |  |  |  |  |  |  |  |  |  |  |  |  |  |  |  |  |  |  | F13H6.18 |  |
|  |  |  |  |  |  |  |  |  |  |  |  |  |  |  |  |  |  |  |  |  |  |  |  |  |  |  |  |  |  |  |  |  |  |  |  |  |  | B0281.7 |  |
|  |  |  |  |  |  |  |  |  |  |  |  |  |  |  |  |  |  |  |  |  |  |  |  |  |  |  |  |  |  |  |  |  |  |  |  |  |  | *ins-8* | INSulin related |
|  |  |  |  |  |  |  |  |  |  |  |  |  |  |  |  |  |  |  |  |  |  |  |  |  |  |  |  |  |  |  |  |  |  |  |  |  |  | F58E2.11 |  |
|  |  |  |  |  |  |  |  |  |  |  |  |  |  |  |  |  |  |  |  |  |  |  |  |  |  |  |  |  |  |  |  |  |  |  |  |  |  | *oac-15* | O-ACyltransferase homolog |
|  |  |  |  |  |  |  |  |  |  |  |  |  |  |  |  |  |  |  |  |  |  |  |  |  |  |  |  |  |  |  |  |  |  |  |  |  |  | F56H9.2 |  |
|  |  |  |  |  |  |  |  |  |  |  |  |  |  |  |  |  |  |  |  |  |  |  |  |  |  |  |  |  |  |  |  |  |  |  |  |  |  | *rpl-25.1* | Ribosomal Protein, Large subunit |
|  |  |  |  |  |  |  |  |  |  |  |  |  |  |  |  |  |  |  |  |  |  |  |  |  |  |  |  |  |  |  |  |  |  |  |  |  |  | *cri-3* | Conserved Regulator of Innate immunity |
|  |  |  |  |  |  |  |  |  |  |  |  |  |  |  |  |  |  |  |  |  |  |  |  |  |  |  |  |  |  |  |  |  |  |  |  |  |  | *cyn-1* | CYclophyliN |
|  |  |  |  |  |  |  |  |  |  |  |  |  |  |  |  |  |  |  |  |  |  |  |  |  |  |  |  |  |  |  |  |  |  |  |  |  |  | Y54G2A.23 |  |
|  |  |  |  |  |  |  |  |  |  |  |  |  |  |  |  |  |  |  |  |  |  |  |  |  |  |  |  |  |  |  |  |  |  |  |  |  |  | Y39A1A.14 |  |
|  |  |  |  |  |  |  |  |  |  |  |  |  |  |  |  |  |  |  |  |  |  |  |  |  |  |  |  |  |  |  |  |  |  |  |  |  |  | *eef-1B.1* | Eukaryotic translation Elongation Factor |
|  |  |  |  |  |  |  |  |  |  |  |  |  |  |  |  |  |  |  |  |  |  |  |  |  |  |  |  |  |  |  |  |  |  |  |  |  |  | *aqp-11* | AQuaPorin or aquaglyceroporin related |
|  |  |  |  |  |  |  |  |  |  |  |  |  |  |  |  |  |  |  |  |  |  |  |  |  |  |  |  |  |  |  |  |  |  |  |  |  |  | F53C11.3 |  |
|  |  |  |  |  |  |  |  |  |  |  |  |  |  |  |  |  |  |  |  |  |  |  |  |  |  |  |  |  |  |  |  |  |  |  |  |  |  | B0272.3 |  |
|  |  |  |  |  |  |  |  |  |  |  |  |  |  |  |  |  |  |  |  |  |  |  |  |  |  |  |  |  |  |  |  |  |  |  |  |  |  | *bmy-1* | Boca/MESD chaperone for YWTD beta-propeller-EGF |
|  |  |  |  |  |  |  |  |  |  |  |  |  |  |  |  |  |  |  |  |  |  |  |  |  |  |  |  |  |  |  |  |  |  |  |  |  |  | T12G3.7 |  |
|  |  |  |  |  |  |  |  |  |  |  |  |  |  |  |  |  |  |  |  |  |  |  |  |  |  |  |  |  |  |  |  |  |  |  |  |  |  | *mrpl-12* | Mitochondrial Ribosomal Protein, Large |
|  |  |  |  |  |  |  |  |  |  |  |  |  |  |  |  |  |  |  |  |  |  |  |  |  |  |  |  |  |  |  |  |  |  |  |  |  |  | *tct-1* | TCTP (translationally-controlled tumor protein) homolog |
|  |  |  |  |  |  |  |  |  |  |  |  |  |  |  |  |  |  |  |  |  |  |  |  |  |  |  |  |  |  |  |  |  |  |  |  |  |  | Y41E3.8 |  |
|  |  |  |  |  |  |  |  |  |  |  |  |  |  |  |  |  |  |  |  |  |  |  |  |  |  |  |  |  |  |  |  |  |  |  |  |  |  | R53.5 |  |
|  |  |  |  |  |  |  |  |  |  |  |  |  |  |  |  |  |  |  |  |  |  |  |  |  |  |  |  |  |  |  |  |  |  |  |  |  |  | *exc-9* | EXCretory canal abnormal |
|  |  |  |  |  |  |  |  |  |  |  |  |  |  |  |  |  |  |  |  |  |  |  |  |  |  |  |  |  |  |  |  |  |  |  |  |  |  | C47B2.9 |  |
|  |  |  |  |  |  |  |  |  |  |  |  |  |  |  |  |  |  |  |  |  |  |  |  |  |  |  |  |  |  |  |  |  |  |  |  |  |  | C06A12.3 |  |
|  |  |  |  |  |  |  |  |  |  |  |  |  |  |  |  |  |  |  |  |  |  |  |  |  |  |  |  |  |  |  |  |  |  |  |  |  |  | *iff-2* | Initiation Factor Five (eIF-5A) homolog |
|  |  |  |  |  |  |  |  |  |  |  |  |  |  |  |  |  |  |  |  |  |  |  |  |  |  |  |  |  |  |  |  |  |  |  |  |  |  | *eef-1B.2* | Eukaryotic translation Elongation Factor |
|  |  |  |  |  |  |  |  |  |  |  |  |  |  |  |  |  |  |  |  |  |  |  |  |  |  |  |  |  |  |  |  |  |  |  |  |  |  | *cyn-5* | CYclophyliN |
|  |  |  |  |  |  |  |  |  |  |  |  |  |  |  |  |  |  |  |  |  |  |  |  |  |  |  |  |  |  |  |  |  |  |  |  |  |  | *mrps-28* | Mitochondrial Ribosomal Protein, Small |
|  |  |  |  |  |  |  |  |  |  |  |  |  |  |  |  |  |  |  |  |  |  |  |  |  |  |  |  |  |  |  |  |  |  |  |  |  |  | *eif-6* | Eukaryotic Initiation Factor |
|  |  |  |  |  |  |  |  |  |  |  |  |  |  |  |  |  |  |  |  |  |  |  |  |  |  |  |  |  |  |  |  |  |  |  |  |  |  | Y54E10A.10 |  |
|  |  |  |  |  |  |  |  |  |  |  |  |  |  |  |  |  |  |  |  |  |  |  |  |  |  |  |  |  |  |  |  |  |  |  |  |  |  | C43E11.9 |  |
|  |  |  |  |  |  |  |  |  |  |  |  |  |  |  |  |  |  |  |  |  |  |  |  |  |  |  |  |  |  |  |  |  |  |  |  |  |  | F53E10.6 |  |
|  |  |  |  |  |  |  |  |  |  |  |  |  |  |  |  |  |  |  |  |  |  |  |  |  |  |  |  |  |  |  |  |  |  |  |  |  |  | M28.5 |  |
|  |  |  |  |  |  |  |  |  |  |  |  |  |  |  |  |  |  |  |  |  |  |  |  |  |  |  |  |  |  |  |  |  |  |  |  |  |  | Y48A6B.3 |  |
|  |  |  |  |  |  |  |  |  |  |  |  |  |  |  |  |  |  |  |  |  |  |  |  |  |  |  |  |  |  |  |  |  |  |  |  |  |  | W04C9.4 |  |
|  |  |  |  |  |  |  |  |  |  |  |  |  |  |  |  |  |  |  |  |  |  |  |  |  |  |  |  |  |  |  |  |  |  |  |  |  |  | T06D8.9 |  |
|  |  |  |  |  |  |  |  |  |  |  |  |  |  |  |  |  |  |  |  |  |  |  |  |  |  |  |  |  |  |  |  |  |  |  |  |  |  | F53A3.7 |  |
|  |  |  |  |  |  |  |  |  |  |  |  |  |  |  |  |  |  |  |  |  |  |  |  |  |  |  |  |  |  |  |  |  |  |  |  |  |  | *rpl-3* | Ribosomal Protein, Large subunit |
|  |  |  |  |  |  |  |  |  |  |  |  |  |  |  |  |  |  |  |  |  |  |  |  |  |  |  |  |  |  |  |  |  |  |  |  |  |  | *rps-6* | Ribosomal Protein, Small subunit |
|  |  |  |  |  |  |  |  |  |  |  |  |  |  |  |  |  |  |  |  |  |  |  |  |  |  |  |  |  |  |  |  |  |  |  |  |  |  | *rps-9* | Ribosomal Protein, Small subunit |
|  |  |  |  |  |  |  |  |  |  |  |  |  |  |  |  |  |  |  |  |  |  |  |  |  |  |  |  |  |  |  |  |  |  |  |  |  |  | *rps-2* | Ribosomal Protein, Small subunit |
|  |  |  |  |  |  |  |  |  |  |  |  |  |  |  |  |  |  |  |  |  |  |  |  |  |  |  |  |  |  |  |  |  |  |  |  |  |  | *rack-1* | RACK1 (mammalian Receptor of Activated C Kinase) homolog |
|  |  |  |  |  |  |  |  |  |  |  |  |  |  |  |  |  |  |  |  |  |  |  |  |  |  |  |  |  |  |  |  |  |  |  |  |  |  | *rpl-7A* | Ribosomal Protein, Large subunit |
|  |  |  |  |  |  |  |  |  |  |  |  |  |  |  |  |  |  |  |  |  |  |  |  |  |  |  |  |  |  |  |  |  |  |  |  |  |  | *rpl-4* | Ribosomal Protein, Large subunit |
|  |  |  |  |  |  |  |  |  |  |  |  |  |  |  |  |  |  |  |  |  |  |  |  |  |  |  |  |  |  |  |  |  |  |  |  |  |  | *rla-0* | Ribosomal protein, Large subunit, Acidic (P1) |
|  |  |  |  |  |  |  |  |  |  |  |  |  |  |  |  |  |  |  |  |  |  |  |  |  |  |  |  |  |  |  |  |  |  |  |  |  |  | *rps-0* | Ribosomal Protein, Small subunit |
|  |  |  |  |  |  |  |  |  |  |  |  |  |  |  |  |  |  |  |  |  |  |  |  |  |  |  |  |  |  |  |  |  |  |  |  |  |  | *rpl-25.2* | Ribosomal Protein, Large subunit |
|  |  |  |  |  |  |  |  |  |  |  |  |  |  |  |  |  |  |  |  |  |  |  |  |  |  |  |  |  |  |  |  |  |  |  |  |  |  | *rps-3* | Ribosomal Protein, Small subunit |
|  |  |  |  |  |  |  |  |  |  |  |  |  |  |  |  |  |  |  |  |  |  |  |  |  |  |  |  |  |  |  |  |  |  |  |  |  |  | *rpl-12* | Ribosomal Protein, Large subunit |
|  |  |  |  |  |  |  |  |  |  |  |  |  |  |  |  |  |  |  |  |  |  |  |  |  |  |  |  |  |  |  |  |  |  |  |  |  |  | *rpl-1* | Ribosomal Protein, Large subunit |
|  |  |  |  |  |  |  |  |  |  |  |  |  |  |  |  |  |  |  |  |  |  |  |  |  |  |  |  |  |  |  |  |  |  |  |  |  |  | *rpl-5* | Ribosomal Protein, Large subunit |
|  |  |  |  |  |  |  |  |  |  |  |  |  |  |  |  |  |  |  |  |  |  |  |  |  |  |  |  |  |  |  |  |  |  |  |  |  |  | *rps-24* | Ribosomal Protein, Small subunit |
|  |  |  |  |  |  |  |  |  |  |  |  |  |  |  |  |  |  |  |  |  |  |  |  |  |  |  |  |  |  |  |  |  |  |  |  |  |  | *rpl-27* | Ribosomal Protein, Large subunit |
|  |  |  |  |  |  |  |  |  |  |  |  |  |  |  |  |  |  |  |  |  |  |  |  |  |  |  |  |  |  |  |  |  |  |  |  |  |  | *rps-18* | Ribosomal Protein, Small subunit |
|  |  |  |  |  |  |  |  |  |  |  |  |  |  |  |  |  |  |  |  |  |  |  |  |  |  |  |  |  |  |  |  |  |  |  |  |  |  | *rps-1* | Ribosomal Protein, Small subunit |
|  |  |  |  |  |  |  |  |  |  |  |  |  |  |  |  |  |  |  |  |  |  |  |  |  |  |  |  |  |  |  |  |  |  |  |  |  |  | *rps-17* | Ribosomal Protein, Small subunit |
|  |  |  |  |  |  |  |  |  |  |  |  |  |  |  |  |  |  |  |  |  |  |  |  |  |  |  |  |  |  |  |  |  |  |  |  |  |  | *rpl-10* | Ribosomal Protein, Large subunit |
|  |  |  |  |  |  |  |  |  |  |  |  |  |  |  |  |  |  |  |  |  |  |  |  |  |  |  |  |  |  |  |  |  |  |  |  |  |  | *rpl-11.2* | Ribosomal Protein, Large subunit |
|  |  |  |  |  |  |  |  |  |  |  |  |  |  |  |  |  |  |  |  |  |  |  |  |  |  |  |  |  |  |  |  |  |  |  |  |  |  | Y37E3.8 |  |
|  |  |  |  |  |  |  |  |  |  |  |  |  |  |  |  |  |  |  |  |  |  |  |  |  |  |  |  |  |  |  |  |  |  |  |  |  |  | *rpl-33* | Ribosomal Protein, Large subunit |
|  |  |  |  |  |  |  |  |  |  |  |  |  |  |  |  |  |  |  |  |  |  |  |  |  |  |  |  |  |  |  |  |  |  |  |  |  |  | *rpl-7* | Ribosomal Protein, Large subunit |
|  |  |  |  |  |  |  |  |  |  |  |  |  |  |  |  |  |  |  |  |  |  |  |  |  |  |  |  |  |  |  |  |  |  |  |  |  |  | *rpl-6* | Ribosomal Protein, Large subunit |
|  |  |  |  |  |  |  |  |  |  |  |  |  |  |  |  |  |  |  |  |  |  |  |  |  |  |  |  |  |  |  |  |  |  |  |  |  |  | *rps-7* | Ribosomal Protein, Small subunit |
|  |  |  |  |  |  |  |  |  |  |  |  |  |  |  |  |  |  |  |  |  |  |  |  |  |  |  |  |  |  |  |  |  |  |  |  |  |  | *rps-13* | Ribosomal Protein, Small subunit |
|  |  |  |  |  |  |  |  |  |  |  |  |  |  |  |  |  |  |  |  |  |  |  |  |  |  |  |  |  |  |  |  |  |  |  |  |  |  | *rps-15* | Ribosomal Protein, Small subunit |
|  |  |  |  |  |  |  |  |  |  |  |  |  |  |  |  |  |  |  |  |  |  |  |  |  |  |  |  |  |  |  |  |  |  |  |  |  |  | *rps-23* | Ribosomal Protein, Small subunit |
|  |  |  |  |  |  |  |  |  |  |  |  |  |  |  |  |  |  |  |  |  |  |  |  |  |  |  |  |  |  |  |  |  |  |  |  |  |  | *rpl-17* | Ribosomal Protein, Large subunit |
|  |  |  |  |  |  |  |  |  |  |  |  |  |  |  |  |  |  |  |  |  |  |  |  |  |  |  |  |  |  |  |  |  |  |  |  |  |  | *rpl-22* | Ribosomal Protein, Large subunit |
|  |  |  |  |  |  |  |  |  |  |  |  |  |  |  |  |  |  |  |  |  |  |  |  |  |  |  |  |  |  |  |  |  |  |  |  |  |  | *rpl-21* | Ribosomal Protein, Large subunit |
|  |  |  |  |  |  |  |  |  |  |  |  |  |  |  |  |  |  |  |  |  |  |  |  |  |  |  |  |  |  |  |  |  |  |  |  |  |  | C37A2.7 |  |
|  |  |  |  |  |  |  |  |  |  |  |  |  |  |  |  |  |  |  |  |  |  |  |  |  |  |  |  |  |  |  |  |  |  |  |  |  |  | *rpl-32* | Ribosomal Protein, Large subunit |
|  |  |  |  |  |  |  |  |  |  |  |  |  |  |  |  |  |  |  |  |  |  |  |  |  |  |  |  |  |  |  |  |  |  |  |  |  |  | *rps-30* | Ribosomal Protein, Small subunit |
|  |  |  |  |  |  |  |  |  |  |  |  |  |  |  |  |  |  |  |  |  |  |  |  |  |  |  |  |  |  |  |  |  |  |  |  |  |  | *rpl-23* | Ribosomal Protein, Large subunit |
|  |  |  |  |  |  |  |  |  |  |  |  |  |  |  |  |  |  |  |  |  |  |  |  |  |  |  |  |  |  |  |  |  |  |  |  |  |  | *rps-14* | Ribosomal Protein, Small subunit |
|  |  |  |  |  |  |  |  |  |  |  |  |  |  |  |  |  |  |  |  |  |  |  |  |  |  |  |  |  |  |  |  |  |  |  |  |  |  | *rpl-13* | Ribosomal Protein, Large subunit |
|  |  |  |  |  |  |  |  |  |  |  |  |  |  |  |  |  |  |  |  |  |  |  |  |  |  |  |  |  |  |  |  |  |  |  |  |  |  | *rpl-31* | Ribosomal Protein, Large subunit |
|  |  |  |  |  |  |  |  |  |  |  |  |  |  |  |  |  |  |  |  |  |  |  |  |  |  |  |  |  |  |  |  |  |  |  |  |  |  | *rps-20* | Ribosomal Protein, Small subunit |
|  |  |  |  |  |  |  |  |  |  |  |  |  |  |  |  |  |  |  |  |  |  |  |  |  |  |  |  |  |  |  |  |  |  |  |  |  |  | *rps-22* | Ribosomal Protein, Small subunit |
|  |  |  |  |  |  |  |  |  |  |  |  |  |  |  |  |  |  |  |  |  |  |  |  |  |  |  |  |  |  |  |  |  |  |  |  |  |  | *rps-8* | Ribosomal Protein, Small subunit |
|  |  |  |  |  |  |  |  |  |  |  |  |  |  |  |  |  |  |  |  |  |  |  |  |  |  |  |  |  |  |  |  |  |  |  |  |  |  | *ubl-1* | UBiquitin-Like family |
|  |  |  |  |  |  |  |  |  |  |  |  |  |  |  |  |  |  |  |  |  |  |  |  |  |  |  |  |  |  |  |  |  |  |  |  |  |  | *rps-5* | Ribosomal Protein, Small subunit |
|  |  |  |  |  |  |  |  |  |  |  |  |  |  |  |  |  |  |  |  |  |  |  |  |  |  |  |  |  |  |  |  |  |  |  |  |  |  | *col-103* | COLlagen |
|  |  |  |  |  |  |  |  |  |  |  |  |  |  |  |  |  |  |  |  |  |  |  |  |  |  |  |  |  |  |  |  |  |  |  |  |  |  | R12E2.13 |  |
|  |  |  |  |  |  |  |  |  |  |  |  |  |  |  |  |  |  |  |  |  |  |  |  |  |  |  |  |  |  |  |  |  |  |  |  |  |  | *rps-12* | Ribosomal Protein, Small subunit |
|  |  |  |  |  |  |  |  |  |  |  |  |  |  |  |  |  |  |  |  |  |  |  |  |  |  |  |  |  |  |  |  |  |  |  |  |  |  | *rps-16* | Ribosomal Protein, Small subunit |
|  |  |  |  |  |  |  |  |  |  |  |  |  |  |  |  |  |  |  |  |  |  |  |  |  |  |  |  |  |  |  |  |  |  |  |  |  |  | *rpia-1* | Ribose 5-Phosphate Isomerase A |
|  |  |  |  |  |  |  |  |  |  |  |  |  |  |  |  |  |  |  |  |  |  |  |  |  |  |  |  |  |  |  |  |  |  |  |  |  |  | *nhr-210* | Nuclear Hormone Receptor family |
|  |  |  |  |  |  |  |  |  |  |  |  |  |  |  |  |  |  |  |  |  |  |  |  |  |  |  |  |  |  |  |  |  |  |  |  |  |  | *srw-99* | Serpentine Receptor, class W |
|  |  |  |  |  |  |  |  |  |  |  |  |  |  |  |  |  |  |  |  |  |  |  |  |  |  |  |  |  |  |  |  |  |  |  |  |  |  | *his-33* | HIStone |
|  |  |  |  |  |  |  |  |  |  |  |  |  |  |  |  |  |  |  |  |  |  |  |  |  |  |  |  |  |  |  |  |  |  |  |  |  |  | *srh-182* | Serpentine Receptor, class H |
|  |  |  |  |  |  |  |  |  |  |  |  |  |  |  |  |  |  |  |  |  |  |  |  |  |  |  |  |  |  |  |  |  |  |  |  |  |  | C18H7.11 |  |
|  |  |  |  |  |  |  |  |  |  |  |  |  |  |  |  |  |  |  |  |  |  |  |  |  |  |  |  |  |  |  |  |  |  |  |  |  |  | T04C12.30 |  |
|  |  |  |  |  |  |  |  |  |  |  |  |  |  |  |  |  |  |  |  |  |  |  |  |  |  |  |  |  |  |  |  |  |  |  |  |  |  | *fbxa-58* | F-box A protein |
|  |  |  |  |  |  |  |  |  |  |  |  |  |  |  |  |  |  |  |  |  |  |  |  |  |  |  |  |  |  |  |  |  |  |  |  |  |  | T15B7.6 |  |
|  |  |  |  |  |  |  |  |  |  |  |  |  |  |  |  |  |  |  |  |  |  |  |  |  |  |  |  |  |  |  |  |  |  |  |  |  |  | F32F2.3 |  |
|  |  |  |  |  |  |  |  |  |  |  |  |  |  |  |  |  |  |  |  |  |  |  |  |  |  |  |  |  |  |  |  |  |  |  |  |  |  | *col-19* | COLlagen |
|  |  |  |  |  |  |  |  |  |  |  |  |  |  |  |  |  |  |  |  |  |  |  |  |  |  |  |  |  |  |  |  |  |  |  |  |  |  | *eif-3.J* | Eukaryotic Initiation Factor |
|  |  |  |  |  |  |  |  |  |  |  |  |  |  |  |  |  |  |  |  |  |  |  |  |  |  |  |  |  |  |  |  |  |  |  |  |  |  | T08D2.1 |  |
|  |  |  |  |  |  |  |  |  |  |  |  |  |  |  |  |  |  |  |  |  |  |  |  |  |  |  |  |  |  |  |  |  |  |  |  |  |  | W09G3.8 |  |
|  |  |  |  |  |  |  |  |  |  |  |  |  |  |  |  |  |  |  |  |  |  |  |  |  |  |  |  |  |  |  |  |  |  |  |  |  |  | F32D8.14 |  |
|  |  |  |  |  |  |  |  |  |  |  |  |  |  |  |  |  |  |  |  |  |  |  |  |  |  |  |  |  |  |  |  |  |  |  |  |  |  | *pdcd-2* | PDCD (mammalian ProgrammeD Cell Death protein) homolog |
|  |  |  |  |  |  |  |  |  |  |  |  |  |  |  |  |  |  |  |  |  |  |  |  |  |  |  |  |  |  |  |  |  |  |  |  |  |  | C23G10.2 |  |
|  |  |  |  |  |  |  |  |  |  |  |  |  |  |  |  |  |  |  |  |  |  |  |  |  |  |  |  |  |  |  |  |  |  |  |  |  |  | K07F5.15 |  |
|  |  |  |  |  |  |  |  |  |  |  |  |  |  |  |  |  |  |  |  |  |  |  |  |  |  |  |  |  |  |  |  |  |  |  |  |  |  | F59A3.4 |  |
|  |  |  |  |  |  |  |  |  |  |  |  |  |  |  |  |  |  |  |  |  |  |  |  |  |  |  |  |  |  |  |  |  |  |  |  |  |  | Y54F10AL.1 |  |
|  |  |  |  |  |  |  |  |  |  |  |  |  |  |  |  |  |  |  |  |  |  |  |  |  |  |  |  |  |  |  |  |  |  |  |  |  |  | *agt-1* | AlkylGuanine DNA alkylTransferase |
|  |  |  |  |  |  |  |  |  |  |  |  |  |  |  |  |  |  |  |  |  |  |  |  |  |  |  |  |  |  |  |  |  |  |  |  |  |  | R04F11.5 |  |
|  |  |  |  |  |  |  |  |  |  |  |  |  |  |  |  |  |  |  |  |  |  |  |  |  |  |  |  |  |  |  |  |  |  |  |  |  |  | T03G11.6 |  |
|  |  |  |  |  |  |  |  |  |  |  |  |  |  |  |  |  |  |  |  |  |  |  |  |  |  |  |  |  |  |  |  |  |  |  |  |  |  | Y106G6H.14 |  |
|  |  |  |  |  |  |  |  |  |  |  |  |  |  |  |  |  |  |  |  |  |  |  |  |  |  |  |  |  |  |  |  |  |  |  |  |  |  | *dpm-3* | Dolichol Phosphate Mannosyltransferase |
|  |  |  |  |  |  |  |  |  |  |  |  |  |  |  |  |  |  |  |  |  |  |  |  |  |  |  |  |  |  |  |  |  |  |  |  |  |  | Y24D9A.10 |  |
|  |  |  |  |  |  |  |  |  |  |  |  |  |  |  |  |  |  |  |  |  |  |  |  |  |  |  |  |  |  |  |  |  |  |  |  |  |  | C39B10.4 |  |
|  |  |  |  |  |  |  |  |  |  |  |  |  |  |  |  |  |  |  |  |  |  |  |  |  |  |  |  |  |  |  |  |  |  |  |  |  |  | H06I04.9 |  |
|  |  |  |  |  |  |  |  |  |  |  |  |  |  |  |  |  |  |  |  |  |  |  |  |  |  |  |  |  |  |  |  |  |  |  |  |  |  | C09H10.10 |  |
|  |  |  |  |  |  |  |  |  |  |  |  |  |  |  |  |  |  |  |  |  |  |  |  |  |  |  |  |  |  |  |  |  |  |  |  |  |  | F53H2.3 |  |

### Phenotypes enriched

|  |  |  |  |
| --- | --- | --- | --- |
| **Group name** | **Number in cluster** | **Enrichment** | **FDR corrected p** |
| pleiotropic defects severe early emb (RNAi) | 36 | 37.02 | 5.69e-42 |
| other abnormality early emb (RNAi) | 37 | 30.05 | 7.80e-40 |
| maternal sterile (RNAi) | 52 | 5.99 | 2.26e-23 |
| hermaphrodite sterile (RNAi) | 52 | 5.99 | 2.26e-23 |
| hermaphrodite fertility reduced (RNAi) | 52 | 5.99 | 2.26e-23 |
| early embryonic lethal (RNAi) | 43 | 5.62 | 2.94e-17 |
| pathogen resistance increased (RNAi) | 15 | 42.50 | 6.67e-17 |
| hermaphrodite fertility variant (RNAi) | 56 | 3.74 | 7.20e-16 |
| sterile (RNAi) | 58 | 3.47 | 3.37e-15 |
| fertility reduced (RNAi) | 58 | 3.46 | 3.94e-15 |
| organism pathogen response variant (RNAi) | 17 | 18.69 | 3.22e-13 |
| reproductive system physiology variant (RNAi) | 60 | 3.00 | 4.58e-13 |
| organ system physiology variant (RNAi) | 60 | 2.99 | 5.26e-13 |
| postembryonic development variant (RNAi) | 53 | 3.34 | 9.69e-13 |
| fertility variant (RNAi) | 59 | 2.97 | 1.59e-12 |
| larval arrest (RNAi) | 49 | 3.52 | 2.99e-12 |
| developmental growth variant (RNAi) | 49 | 3.52 | 3.02e-12 |
| larval growth variant (RNAi) | 49 | 3.51 | 3.05e-12 |
| larval development variant (RNAi) | 50 | 3.32 | 1.19e-11 |
| growth variant (RNAi) | 62 | 2.51 | 3.63e-10 |
| protein aggregation variant (RNAi) | 25 | 6.35 | 7.02e-10 |
| protein interaction variant (RNAi) | 25 | 6.31 | 7.89e-10 |
| protein protein interaction variant (RNAi) | 25 | 6.31 | 7.89e-10 |
| transgene subcellular localization variant (RNAi) | 26 | 5.59 | 3.46e-09 |
| pattern of transgene expression variant (RNAi) | 35 | 3.75 | 1.73e-08 |
| transgene expression variant (RNAi) | 38 | 3.28 | 8.58e-08 |
| gene expression variant (RNAi) | 38 | 2.97 | 1.27e-06 |
| organism development variant (RNAi) | 66 | 1.94 | 3.21e-06 |
| embryonic lethal (RNAi) | 56 | 2.13 | 6.04e-06 |
| sick (RNAi) | 23 | 4.38 | 6.07e-06 |
| organism physiology variant (RNAi) | 71 | 1.82 | 6.22e-06 |
| embryonic development variant (RNAi) | 56 | 2.11 | 7.49e-06 |
| organism homeostasis metabolism variant (RNAi) | 68 | 1.85 | 1.18e-05 |
| development variant (RNAi) | 67 | 1.84 | 2.00e-05 |
| lethal (RNAi) | 58 | 1.98 | 3.50e-05 |
| molt defect (RNAi) | 14 | 5.78 | 3.02e-04 |
| organism metabolism processing variant (RNAi) | 41 | 2.27 | 3.23e-04 |
| physiology variant (RNAi) | 73 | 1.60 | 8.12e-04 |
| slow growth (RNAi) | 35 | 2.15 | 8.52e-03 |
| organism environmental stimulus response variant (RNAi) | 19 | 3.11 | 1.28e-02 |
| Variant (RNAi) | 79 | 1.39 | 3.96e-02 |

### Anatomy terms enriched

none found

### GO terms enriched

|  |  |  |
| --- | --- | --- |
| **GO term** | **Number of genes** | **FDR-corrected p-value** |
| ribosome | 44 | 2.6e-53 |
| structural constituent of ribosome | 41 | 2.7e-53 |
| translation | 39 | 2.0e-40 |
| non-membrane-bounded organelle | 49 | 8.5e-24 |
| macromolecular complex | 49 | 3.8e-19 |
| cytoplasm | 57 | 2.7e-16 |
| macromolecule biosynthetic process | 46 | 3.1e-16 |
| protein metabolic process | 47 | 4.9e-15 |
| cellular biosynthetic process | 46 | 8.8e-13 |
| apoptotic process | 26 | 5.7e-11 |
| cytosolic small ribosomal subunit | 8 | 7.0e-11 |
| nematode larval development | 50 | 8.7e-11 |
| post-embryonic development | 50 | 1.2e-10 |
| cellular macromolecule metabolic process | 51 | 2.1e-10 |
| reproduction | 54 | 9.4e-10 |
| cell death | 26 | 1.1e-09 |
| growth | 23 | 1.4e-09 |
| intracellular | 63 | 4.9e-09 |
| small ribosomal subunit | 7 | 2.5e-08 |
| intracellular organelle | 33 | 7.8e-06 |
| RNA binding | 15 | 8.1e-06 |
| cell | 63 | 1.3e-05 |
| embryo development ending in birth or egg hatching | 49 | 4.5e-05 |
| molting cycle, collagen and cuticulin-based cuticle | 14 | 1.5e-04 |
| organic substance metabolic process | 53 | 2.6e-04 |
| positive regulation of growth rate | 31 | 2.9e-04 |
| translational elongation | 5 | 2.9e-04 |
| regulation of growth | 34 | 4.9e-04 |
| cytosol | 9 | 5.8e-04 |
| rRNA processing | 5 | 2.6e-03 |
| positive regulation of biological process | 35 | 2.7e-03 |
| multicellular organismal process | 60 | 6.4e-03 |
| anatomical structure development | 40 | 1.0e-02 |
| intracellular organelle part | 25 | 1.9e-02 |
| ribonucleoprotein complex subunit organization | 4 | 2.9e-02 |
| ncRNA metabolic process | 6 | 3.6e-02 |
| cellular component biogenesis | 13 | 4.5e-02 |
| ribosome biogenesis | 3 | 4.9e-02 |

### Expression clusters enriched

|  |  |  |  |
| --- | --- | --- | --- |
| **Group name** | **Number in cluster** | **Enrichment** | **FDR corrected p** |
| The cluster contains genes that are significantly enriched in L1 muscle. | 63 | 5.56 | 1.51e-29 |
| Genes in the top 10% of expression level across the triplicate L3 samples. To generate the top10 and bottom10 gene sets, authors ranked all genes by mean expression array signal intensity across the three replicates, then took the top and bottom deciles (1,841 genes each) to represent genes with high and low expression. | 67 | 4.64 | 2.42e-27 |
| C-lineage related expression profile. WBPaper00025032:cluster\_10 | 23 | 33.32 | 5.87e-26 |
| Genes up or down regulated by 10e-05M of estrogen. The normalized values used were G/R ratio > 2.6 for up-regulation and G/R ratio < 0.38 for down-regulation, which corresponds to 1.39 and -1.39 log(base2) G/R ratio, respectively. | 51 | 4.93 | 3.57e-20 |
| Genes that are up or down regulated by more than 2.1 fold with the t-test p-value less than 0.01 are included in this cluster. | 47 | 4.87 | 5.75e-18 |
| Genes up or down regulated by 10e-09M of cholesterol . The normalized values used were G/R ratio > 2.6 for up-regulation and G/R ratio < 0.38 for down-regulation, which corresponds to 1.39 and -1.39 log(base2) G/R ratio, respectively. | 55 | 3.92 | 1.62e-17 |
| Genome-wide analysis of developmental and sex-regulated gene expression profile. cgc4489\_group\_3 | 38 | 6.24 | 2.46e-17 |
| Early embryonic development gene expression profile. [cgc5767]:cluster\_2 | 29 | 8.72 | 2.32e-16 |
| C-lineage related expression profile. WBPaper00025032:cluster\_25 | 14 | 39.67 | 3.06e-16 |
| Differentially expressed genes during worm lifespan. Medoid 7 Fig.4. | 26 | 9.99 | 7.65e-16 |
| Caenorhabditis elegans Genes with expression levels changed significantly after treatment of Serratia marcescens. | 73 | 2.63 | 1.91e-15 |
| Genes up or down regulated by 10e-05M of progesterone. The normalized values used were G/R ratio > 2.6 for up-regulation and G/R ratio < 0.38 for down-regulation, which corresponds to 1.39 and -1.39 log(base2) G/R ratio, respectively. | 60 | 3.19 | 2.65e-15 |
| Maternal-embryonic class (ME): genes that are in the intersection of the maternal and embryonic classes. | 62 | 2.99 | 1.28e-14 |
| Caenorhabditis elegans Genes with expression levels changed significantly after treatment of Bacillus thurigiensis DB27. | 80 | 2.13 | 2.82e-12 |
| Embryonic (E) subclasses are based on the earliest significant increase(abbreviated pi for primary increase). [cgc5767]:expression\_class\_E\_pi(66\_min) | 20 | 9.11 | 5.07e-11 |
| Embryonic class (E): genes that significantly increase in abundance at some point during embryogenesis. | 66 | 2.36 | 7.57e-11 |
| Genes with decreased expression levels (P < 0.05) under ifg-1 inhibition. | 10 | 35.42 | 1.20e-10 |
| Early embryonic development gene expression profile. [cgc5767]:cluster\_16 | 9 | 39.57 | 6.30e-10 |
| Genes enriched in muscle. | 22 | 6.78 | 9.13e-10 |
| Genes enriched in intestine. | 45 | 2.86 | 9.95e-09 |
| Genes up or down regulated by 10e-09M of testosterone. The normalized values used were G/R ratio > 2.6 for up-regulation and G/R ratio < 0.38 for down-regulation, which corresponds to 1.39 and -1.39 log(base2) G/R ratio, respectively. | 61 | 2.09 | 2.25e-07 |
| Genes up or down regulated by 10e-07M of progesterone. The normalized values used were G/R ratio > 2.6 for up-regulation and G/R ratio < 0.38 for down-regulation, which corresponds to 1.39 and -1.39 log(base2) G/R ratio, respectively. | 57 | 2.14 | 5.48e-07 |
| Caenorhabditis elegans Genes with expression levels changed significantly after treatment of Xenorhabdus nematophila. | 88 | 1.61 | 6.11e-07 |
| Embryonic (E) subclasses are based on the earliest significant increase(abbreviated pi for primary increase). [cgc5767]:expression\_class\_E\_pi(53\_min) | 25 | 3.68 | 6.29e-06 |
| C-lineage related expression profile. WBPaper00025032:cluster\_120 | 5 | 57.96 | 6.43e-06 |
| C-lineage related expression profile. WBPaper00025032:cluster\_2 | 13 | 7.27 | 1.37e-05 |
| Maternal class (M): genes that are called present in at least one of the three PC6 replicates. | 74 | 1.62 | 7.33e-05 |
| Potental DAF-12 target genes identified by ChIP-chip analysis performed on strain ALF4 [daf-12 | 47 | 1.92 | 7.51e-04 |
| Genes that show selective expression in a subset of cell types vs broadly expressed in many cell types. Correspond to 20% - 57% of enriched\_genes for a given cell type. WBPaper00037950:PVD-OLL-neurons\_larva\_SelectivelyEnriched | 13 | 4.64 | 1.54e-03 |
| Developmentally modulated gene cluster. cgc4386\_cluster\_1\_4 | 7 | 8.75 | 4.55e-03 |
| intestinal (adult); 1.2e-04(1.11x). | 13 | 4.03 | 5.92e-03 |
| intestinal (adult); 8.3e-05(1.12x). | 13 | 4.02 | 6.06e-03 |
| intestinal (adult); 3.7e-05(1.14x). topomap mount 7; 5.4e-04(1.54x). | 14 | 3.75 | 6.24e-03 |
| intestinal (adult); 4.5e-05(1.13x). topomap mount 7; 5.6e-04(1.54x). | 14 | 3.74 | 6.36e-03 |
| Early embryonic development gene expression profile. [cgc5767]:cluster\_5 | 8 | 6.85 | 6.41e-03 |
| Genes enriched in HLH-1 heat shock dataset. | 36 | 1.98 | 7.77e-03 |
| Significantly differentially expressed genes after different bacteria treatment. Transcriptional responses of wild-type C. elegans adults were assayed after growth on each of the four bacteria - E. coli, M. luteus, Pseudomonas sp., or B. megaterium. These genes were differentially expressed and statistically significant with multiple testing correction (q < 0.01) across all pair-wise comparisons. | 9 | 5.74 | 7.79e-03 |
| Proteins that showed decreased expression 4h after infection with S. aureus. | 4 | 18.89 | 1.41e-02 |
| Genes expressed in embryonic motor neurons (identified by unc-4::GFP expressing cells). | 69 | 1.45 | 1.49e-02 |
| Genes downregulated in daf-2(-) compared to daf-16(-); daf-2(-) | 10 | 4.47 | 2.03e-02 |
| Embryonic (E) subclasses are based on the earliest significant increase(abbreviated pi for primary increase). [cgc5767]:expression\_class\_E\_pi(83\_min) | 12 | 3.72 | 2.25e-02 |
| Class A gene expression showed down regulation in lin-14(lf) in L1, no change in lin-4(lf) in L2. | 7 | 6.38 | 2.74e-02 |
| Clr; 7.7e-04(7.48x). osmoregulation; 8.8e-04(8.34x). | 3 | 27.32 | 3.64e-02 |
| Jiang M. et al. group 3; 6.0e-04(2.10x). | 5 | 9.24 | 4.50e-02 |
| body wall muscle (adult); 6.4e-04(1.54x). Jiang M. et al. group 3; 7.2e-04(2.07x). ex). cell (adult); 8.9e-04(2.31x). | 5 | 9.11 | 4.75e-02 |

### Motifs enriched

|  |  |  |  |  |  |
| --- | --- | --- | --- | --- | --- |
| **Motif** | **Logo** | **Possible orthologs** | **Number of motifs in cluster** | **Enrichment** | **FDR corrected p** |
| pTH8649 |  | mbr-1 (-0.72) | 53 | 2.54 | 1.1e-08 |
| Mv109 |  | pax-2 | 39 | 2.67 | 2.5e-06 |
| pTH2933 |  | F58G1.2 | 39 | 2.53 | 9.3e-06 |
| MA0146.2 |  | F58G1.2 | 43 | 2.30 | 2.0e-05 |
| Dll\_Cell\_FBgn0000157 |  | ceh-43 | 76 | 1.57 | 1.1e-04 |
| pTH8916 |  | hmg-12 lin-39 Y116A8C.22 | 86 | 1.42 | 6.2e-04 |
| pTH9220 |  | mbr-1 (-0.72) | 43 | 1.97 | 7.3e-04 |
| PAX5\_si |  | pax-2 D1081.8 | 15 | 3.99 | 1.0e-03 |
| pTH9049 |  | ztf-2 | 69 | 1.54 | 1.2e-03 |
| FOXJ3\_si |  | lin-31 | 95 | 1.33 | 1.2e-03 |
| Barhl1\_3 |  | ceh-31 | 75 | 1.48 | 1.2e-03 |
| sqz\_SOLEXA\_5\_FBgn0010768 |  | lin-29 fkh-7 mel-28 | 80 | 1.44 | 1.4e-03 |
| V$PAX5\_01 |  | pax-2 | 52 | 1.74 | 1.5e-03 |
| NR2E3\_f1 |  | nhr-100 lin-1 | 90 | 1.35 | 1.7e-03 |
| pTH9335 |  | mel-28 | 76 | 1.46 | 1.7e-03 |
| pTH5059 |  | lin-22 | 41 | 1.93 | 2.0e-03 |
| Zfp161\_2858 |  | pzf-1 | 44 | 1.86 | 2.0e-03 |
| TBX2\_1 |  | tbx-39 tbx-42 | 38 | 1.98 | 2.5e-03 |
| MA0041.1 |  | let-381 | 94 | 1.31 | 2.7e-03 |
| pTH9097 |  | Y116A8C.22 | 63 | 1.56 | 2.8e-03 |
| MA0537.1 |  | blmp-1 | 95 | 1.30 | 2.8e-03 |
| pTH10797 |  | K11D2.4 lin-29 | 82 | 1.39 | 3.3e-03 |
| pTH9056 |  | mbr-1 (-0.72) nhr-177 bed-3 | 36 | 1.99 | 3.8e-03 |
| MA0028.1 |  | lin-1 | 68 | 1.49 | 3.8e-03 |
| MA0049.1 |  | hbl-1 | 77 | 1.42 | 3.9e-03 |
| pTH9082 |  | mab-23 | 80 | 1.39 | 4.0e-03 |
| pTH9380 |  | mel-28 | 63 | 1.54 | 4.1e-03 |
| HXD4\_f1 |  | lin-39 | 80 | 1.39 | 4.3e-03 |
| HMGA1\_f1 |  | let-381 mel-28 Y116A8C.22 Y61A9LA.9 | 83 | 1.37 | 4.5e-03 |
| ONECUT2\_1 |  | ceh-48 (-0.74) | 36 | 1.96 | 4.5e-03 |
| pTH9306 |  | lsl-1 | 17 | 3.03 | 5.5e-03 |
| pTH9242 |  | mel-28 | 93 | 1.29 | 6.6e-03 |
| pTH9260 |  | mel-28 | 90 | 1.31 | 7.5e-03 |
| MA0497.1 |  | mef-2 | 87 | 1.32 | 7.6e-03 |
| pTH9951 |  | mex-6 | 60 | 1.53 | 8.0e-03 |
| MA0161.1 |  | nfi-1 F49E12.6 | 37 | 1.87 | 8.4e-03 |
| pTH9709 |  | die-1 | 82 | 1.35 | 8.4e-03 |
| ARI3A\_do |  | cfi-1 | 54 | 1.59 | 8.6e-03 |
| Six1\_0935 |  | ceh-32 (-0.52) | 12 | 3.74 | 1.0e-02 |
| pTH10837 |  | T22H9.4 | 77 | 1.38 | 1.0e-02 |
| pTH3046 |  | Y116A8C.22 | 58 | 1.53 | 1.1e-02 |
| pTH9189 |  | dmd-3 (0.55) ceh-18 | 82 | 1.34 | 1.1e-02 |
| pTH6143 |  | pal-1 php-3 | 80 | 1.35 | 1.2e-02 |
| MA0470.1 |  | efl-1 F49E12.6 | 36 | 1.86 | 1.2e-02 |
| pnr\_SANGER\_5\_FBgn0003117 |  | elt-1 | 86 | 1.31 | 1.2e-02 |
| tgo\_ss\_SANGER\_5\_FBgn0015014 |  | aha-1 ahr-1 | 47 | 1.65 | 1.3e-02 |
| Mafk\_3106 |  | F45H11.6 | 73 | 1.39 | 1.4e-02 |
| cad\_FlyReg\_FBgn0000251 |  | ceh-13 | 45 | 1.67 | 1.5e-02 |
| Eip74EF\_FlyReg\_FBgn0000567 |  | C24A1.2 | 79 | 1.35 | 1.5e-02 |
| ZBTB49\_1 |  | C46E10.9 | 55 | 1.53 | 1.6e-02 |
| ERG\_4 |  | lin-1 | 45 | 1.65 | 1.7e-02 |
| MA0536.1 |  | elt-1 | 19 | 2.50 | 1.7e-02 |
| ECC-1\_ERALPHA\_HudsonAlpha |  | nhr-71 | 36 | 1.80 | 1.9e-02 |
| MA0198.1 |  | alr-1 egl-5 | 65 | 1.43 | 2.0e-02 |
| ELK4\_1 |  | lin-1 | 58 | 1.49 | 2.1e-02 |
| pTH9089 |  | ref-2 | 41 | 1.69 | 2.2e-02 |
| MSX2\_f1 |  | ceh-1 | 67 | 1.40 | 2.4e-02 |
| pTH10696 |  | Y44A6D.3 | 32 | 1.86 | 2.5e-02 |
| pTH4325 |  | ceh-18 | 70 | 1.38 | 2.5e-02 |
| pTH10040 |  | slr-2 | 63 | 1.43 | 2.5e-02 |
| MA0262.1 |  | mab-3 hsf-1 | 69 | 1.38 | 2.6e-02 |
| MAX\_1 |  | mxl-1 | 17 | 2.55 | 2.7e-02 |
| pTH9254 |  | mel-28 | 89 | 1.27 | 2.7e-02 |
| pTH5169 |  | cfi-1 | 83 | 1.30 | 2.9e-02 |
| pTH9173 |  | efl-2 | 40 | 1.67 | 3.0e-02 |
| MA0032.1 |  | let-381 | 20 | 2.26 | 3.5e-02 |
| MA0538.1 |  | daf-12 | 56 | 1.46 | 3.6e-02 |
| pTH7875 |  | mel-28 | 63 | 1.41 | 3.7e-02 |
| MA0544.1 |  | gei-11 | 47 | 1.55 | 3.8e-02 |
| FOXI1\_f1 |  | lin-31 | 63 | 1.40 | 3.9e-02 |
| MA0453.1 |  | ceh-18 | 78 | 1.31 | 4.0e-02 |
| TBP\_f1 |  | tbp-1 | 70 | 1.35 | 4.3e-02 |
| SMAD3\_1 |  | daf-8 | 20 | 2.21 | 4.3e-02 |
| pTH9219 |  | C01B12.2 | 6 | 5.74 | 4.3e-02 |
| Ara\_Cell\_FBgn0015904 |  | irx-1 | 78 | 1.30 | 4.6e-02 |
| exd\_FlyReg\_FBgn0000611 |  | ceh-20 | 90 | 1.24 | 4.9e-02 |
| PO4F2\_si |  | unc-86 | 62 | 1.40 | 4.9e-02 |

### Correlated (and anti-correlated) transcription factors

|  |  |
| --- | --- |
| **Transcription factor** | **Correlation** |
| nhr-122 | 0.77 |
| nhr-104 | 0.60 |
| grh-1 | 0.59 |
| nhr-204 | 0.58 |
| hnd-1 | 0.57 |
| dmd-3 | 0.55 |
| C02F12.5 | 0.55 |
| nhr-13 | 0.55 |
| nhr-64 | 0.54 |
| icd-2 | 0.51 |
| duxl-1 | 0.51 |
| nhr-146 | 0.51 |
| hlh-12 | 0.50 |
| F27D4.4 | 0.50 |
| ztf-27 | 0.49 |
| ceh-82 | 0.49 |
| K02D7.2 | 0.48 |
| nhr-90 | 0.48 |
| nhr-168 | 0.48 |
| C30G4.7 | 0.47 |
| odd-2 | 0.47 |
| ces-2 | 0.47 |
| nhr-70 | 0.46 |
| ref-1 | 0.46 |
| nhr-112 | 0.45 |
| ctbp-1 | -0.74 |
| ast-1 | -0.74 |
| dhhc-14 | -0.76 |
| Y47G6A.7 | -0.76 |
| sox-4 | -0.77 |
| daf-3 | -0.77 |
| nhr-26 | -0.77 |
| saeg-2 | -0.77 |
| lim-4 | -0.77 |
| sem-4 | -0.78 |
| Y17G7B.22 | -0.78 |
| C02F5.12 | -0.79 |
| daf-19 | -0.79 |
| ceh-62 | -0.80 |
| gei-3 | -0.80 |
| ceh-54 | -0.82 |
| unc-42 | -0.82 |
| ceh-58 | -0.82 |
| C33H5.17 | -0.83 |
| aptf-4 | -0.83 |
| C34F6.9 | -0.84 |
| dmd-10 | -0.84 |
| nhr-47 | -0.84 |
| C17E4.6 | -0.86 |
| mgl-2 | -0.89 |

### ChIP peaks enriched

|  |  |  |  |  |
| --- | --- | --- | --- | --- |
| **Gene** | **Experiment** | **Number of upstream peaks** | **Enrichment** | **FDR corrected p** |
| lsy-2 | LSY-2\_Larvae-L2-stage | 65 | 6.88 | 1.7e-36 |
| F16B12.6 | F16B12.6\_Fed-L1-stage-larvae | 61 | 6.26 | 2.0e-31 |
| nhr-237 | NHR-237\_Embryos | 58 | 6.40 | 5.2e-30 |
| R02D3.7 | R02D3.7\_Larvae-L2-stage | 63 | 5.56 | 8.0e-30 |
| nhr-77 | NHR-77\_Fed-L1-stage-larvae | 82 | 3.72 | 9.9e-30 |
| efl-1 | EFL-1\_Young-adult | 74 | 4.30 | 1.5e-29 |
| ceh-39 | CEH-39\_Embryos | 65 | 5.23 | 1.7e-29 |
| nhr-237 | NHR-237\_Larvae-L1-stage | 48 | 8.54 | 2.7e-29 |
| dpl-1 | DPL-1\_Young-adult | 78 | 3.90 | 6.4e-29 |
| F23B12.7 | F23B12.7\_Young-adult | 66 | 4.95 | 1.0e-28 |
| lin-15 | LIN-15B\_Fed-L1-stage-larvae | 63 | 5.31 | 1.1e-28 |
| ztf-7 | ZTF-7\_Larvae-L4-stage | 65 | 4.93 | 4.5e-28 |
| R02D3.7 | R02D3.7\_Larvae-L3-stage | 84 | 3.40 | 5.3e-28 |
| aly-2 | ALY-2\_Fed-L1-stage-larvae | 71 | 4.32 | 5.6e-28 |
| fos-1 | FOS-1\_Fed-L1-stage-larvae | 79 | 3.69 | 7.1e-28 |
| lsy-2 | LSY-2\_Embryos | 70 | 4.35 | 1.3e-27 |
| lin-35 | LIN-35\_Fed-L1-stage-larvae | 68 | 4.40 | 7.0e-27 |
| lin-13 | LIN-13\_Larvae-L4-stage | 61 | 5.14 | 8.4e-27 |
| efl-1 | EFL-1\_Larvae-L1-stage | 81 | 3.41 | 1.5e-26 |
| C16A3.4 | C16A3.4\_Fed-L1-stage-larvae | 69 | 4.23 | 2.3e-26 |
| lin-13 | LIN-13\_Larvae-L2-stage | 67 | 4.26 | 1.6e-25 |
| nhr-23 | NHR-23\_Larvae-L3-stage | 75 | 3.63 | 2.4e-25 |
| R02D3.7 | R02D3.7\_Larvae-L4-stage | 54 | 5.72 | 3.5e-25 |
| lsy-2 | LSY-2\_Fed-L1-stage-larvae | 77 | 3.48 | 3.6e-25 |
| F45C12.2 | F45C12.2\_Fed-L1-stage-larvae | 73 | 3.70 | 7.7e-25 |
| nhr-6 | NHR-6\_Larvae-L4-stage | 60 | 4.80 | 1.0e-24 |
| skn-1 | SKN-1\_Larvae-L3-stage | 52 | 5.86 | 1.6e-24 |
| egl-5 | EGL-5\_Larvae-L3-stage | 66 | 4.18 | 1.6e-24 |
| efl-1 | EFL-1\_Fed-L1-stage-larvae | 74 | 3.58 | 1.8e-24 |
| nhr-25 | NHR-25\_Larvae-L2-stage | 71 | 3.76 | 2.7e-24 |
| nfya-1 | NFYA-1\_Late-Embryos | 74 | 3.53 | 4.2e-24 |
| ham-1 | HAM-1\_Larvae-L4-stage | 80 | 3.19 | 4.3e-24 |
| dpl-1 | DPL-1\_Fed-L1-stage-larvae | 74 | 3.52 | 5.4e-24 |
| nfya-1 | NFYA-1\_Young-adult | 43 | 7.66 | 5.7e-24 |
| C01B12.2 | C01B12.2\_Larvae-L2-stage | 89 | 2.76 | 7.5e-24 |
| gei-11 | GEI-11\_Larvae-L2-stage | 67 | 3.97 | 8.5e-24 |
| lsy-2 | LSY-2\_Larvae-L4-stage | 50 | 5.97 | 9.9e-24 |
| nfya-1 | NFYA-1\_Larvae-L3-stage | 69 | 3.78 | 1.7e-23 |
| ceh-38 | CEH-38\_Larvae-L3-stage | 72 | 3.54 | 3.5e-23 |
| zag-1 | ZAG-1\_Fed-L1-stage-larvae | 48 | 6.15 | 3.7e-23 |
| lin-15 | LIN-15B\_Larvae-L4-stage | 41 | 7.84 | 4.4e-23 |
| C34F6.9 | C34F6.9\_Larvae-L2-stage | 77 | 3.19 | 1.0e-22 |
| pax-1 | PAX-1\_Embryos | 39 | 8.32 | 1.0e-22 |
| eor-1 | EOR-1\_Larvae-L3-stage | 77 | 3.11 | 5.6e-22 |
| lin-13 | LIN-13\_Larvae-L1-stage | 40 | 7.59 | 6.1e-22 |
| gei-11 | GEI-11\_Fed-L1-stage-larvae | 73 | 3.30 | 7.4e-22 |
| F45C12.2 | F45C12.2\_Larvae-L2-stage | 43 | 6.58 | 1.9e-21 |
| jun-1 | JUN-1\_Larvae-L4-stage | 59 | 4.23 | 2.0e-21 |
| dve-1 | DVE-1\_Late-Embryos | 66 | 3.54 | 1.4e-20 |
| aly-2 | ALY-2\_Larvae-L3-stage | 50 | 4.89 | 5.1e-20 |
| jun-1 | JUN-1\_Larvae-L1-stage | 63 | 3.64 | 7.0e-20 |
| elt-3 | ELT-3\_Embryos | 57 | 4.10 | 7.2e-20 |
| nhr-77 | NHR-77\_Larvae-L3-stage | 60 | 3.82 | 1.1e-19 |
| gei-11 | GEI-11\_Larvae-L3-stage | 71 | 3.12 | 1.5e-19 |
| lsy-2 | LSY-2\_Larvae-L1-stage | 79 | 2.75 | 2.0e-19 |
| hpl-2 | HPL-2\_Fed-L1-stage-larvae | 79 | 2.73 | 3.1e-19 |
| dpl-1 | DPL-1\_Larvae-L4-stage | 82 | 2.61 | 4.0e-19 |
| pes-1 | PES-1\_Larvae-L4-stage | 72 | 3.00 | 5.4e-19 |
| zag-1 | ZAG-1\_Larvae-L2-stage | 56 | 3.95 | 1.1e-18 |
| ces-1 | CES-1\_Embryos | 71 | 2.98 | 2.1e-18 |
| ham-1 | HAM-1\_Fed-L1-stage-larvae | 70 | 3.00 | 3.4e-18 |
| gei-11 | GEI-11\_Young-adult | 48 | 4.63 | 4.3e-18 |
| lin-35 | LIN-35\_Starved-L1-stage-larvae | 44 | 5.15 | 5.2e-18 |
| nhr-77 | NHR-77\_Larvae-L4-stage | 84 | 2.40 | 1.6e-17 |
| nhr-129 | NHR-129\_Larvae-L2-stage | 81 | 2.49 | 1.9e-17 |
| alr-1 | ALR-1\_Larvae-L2-stage | 64 | 3.19 | 2.4e-17 |
| jun-1 | JUN-1\_Larvae-L3-stage | 53 | 3.94 | 2.5e-17 |
| dve-1 | DVE-1\_Larvae-L4-stage | 56 | 3.69 | 2.5e-17 |
| unc-62 | UNC-62\_Fed-L1-stage-larvae | 30 | 8.50 | 3.1e-17 |
| W03F9.2 | W03F9.2\_L4-Young-Adult-stage-larvae | 87 | 2.28 | 5.1e-17 |
| zag-1 | ZAG-1\_Larvae-L4-stage | 49 | 4.24 | 5.3e-17 |
| nhr-76 | NHR-76\_Larvae-L4-stage | 48 | 4.30 | 8.1e-17 |
| hlh-30 | HLH-30\_Late-Embryos | 40 | 5.39 | 9.2e-17 |
| unc-62 | UNC-62\_Day-Four-Young-Adult | 53 | 3.82 | 9.3e-17 |
| unc-62 | UNC-62\_Young-adult-Day-4 | 53 | 3.82 | 9.3e-17 |
| F45C12.2 | F45C12.2\_Larvae-L3-stage | 43 | 4.90 | 9.9e-17 |
| gei-11 | GEI-11\_Embryos | 37 | 5.89 | 1.5e-16 |
| pha-4 | PHA-4\_Young-adult | 45 | 4.56 | 1.7e-16 |
| elt-1 | ELT-1\_Larvae-L3-stage | 41 | 5.10 | 2.0e-16 |
| ceh-16 | CEH-16\_Larvae-L2-stage | 39 | 5.42 | 2.3e-16 |
| nhr-77 | NHR-77\_Larvae-L2-stage | 40 | 5.21 | 2.9e-16 |
| pha-4 | PHA-4\_Larvae-L2-stage | 72 | 2.68 | 3.2e-16 |
| sem-4 | SEM-4\_Larvae-L2-stage | 67 | 2.88 | 3.8e-16 |
| ztf-4 | ZTF-4\_Larvae-L3-stage | 30 | 7.56 | 7.2e-16 |
| hlh-30 | HLH-30\_Larvae-L4-stage | 48 | 4.05 | 8.4e-16 |
| fos-1 | FOS-1\_Larvae-L2-stage | 75 | 2.50 | 1.9e-15 |
| zag-1 | ZAG-1\_Larvae-L3-stage | 39 | 5.01 | 2.9e-15 |
| fos-1 | FOS-1\_Larvae-L3-stage | 54 | 3.35 | 9.6e-15 |
| sax-3 | SAX-3\_Larvae-L4-stage | 69 | 2.56 | 3.5e-14 |
| mab-5 | MAB-5\_Larvae-L2-stage | 40 | 4.46 | 4.6e-14 |
| ces-1 | CES-1\_Fed-L1-stage-larvae | 38 | 4.66 | 7.7e-14 |
| nhr-11 | NHR-11\_Larvae-L2-stage | 33 | 5.29 | 2.9e-13 |
| ceh-26 | CEH-26\_Late-Embryonic-stage | 48 | 3.40 | 5.9e-13 |
| sax-3 | SAX-3\_Larvae-L2-stage | 51 | 3.21 | 5.9e-13 |
| nhr-28 | NHR-28\_Larvae-L3-stage | 25 | 7.12 | 1.8e-12 |
| ztf-4 | ZTF-4\_Larvae-L2-stage | 33 | 4.87 | 2.6e-12 |
| nhr-6 | NHR-6\_Larvae-L2-stage | 58 | 2.71 | 4.2e-12 |
| pha-4 | PHA-4\_Larvae-L4-stage | 46 | 3.19 | 2.6e-11 |
| nhr-28 | NHR-28\_Larvae-L4-stage | 68 | 2.23 | 6.4e-11 |
| ces-1 | CES-1\_Larvae-L4-stage | 33 | 4.14 | 1.9e-10 |
| nhr-237 | NHR-237\_Larvae-L2-stage | 17 | 9.95 | 2.0e-10 |
| sax-3 | SAX-3\_Fed-L1-stage-larvae | 26 | 5.26 | 3.8e-10 |
| lin-35 | LIN-35\_Young-adult | 36 | 3.67 | 4.6e-10 |
| aha-1 | AHA-1\_Larvae-L4-stage | 25 | 5.35 | 7.1e-10 |
| ces-1 | CES-1\_Larvae-L3-stage | 32 | 4.03 | 8.6e-10 |
| fos-1 | FOS-1\_Larvae-L4-stage | 32 | 3.93 | 1.6e-09 |
| nhr-76 | NHR-76\_Larvae-L3-stage | 30 | 4.16 | 1.9e-09 |
| nhr-21 | NHR-21\_Larvae-L2-stage | 25 | 5.04 | 2.4e-09 |
| unc-62 | UNC-62\_Larvae-L3-stage | 38 | 3.21 | 4.5e-09 |
| ceh-38 | CEH-38\_Larvae-L4-stage | 36 | 3.31 | 7.1e-09 |
| aha-1 | AHA-1\_Fed-L1-stage-larvae | 18 | 6.87 | 1.5e-08 |
| nhr-10 | NHR-10\_Larvae-L4-stage | 20 | 4.93 | 3.3e-07 |
| sea-2 | SEA-2\_Larvae-L3-stage | 22 | 4.23 | 7.9e-07 |
| ztf-4 | ZTF-4\_Larvae-L1-stage | 17 | 5.50 | 1.1e-06 |
| mab-5 | MAB-5\_Embryos | 17 | 5.47 | 1.1e-06 |
| daf-12 | DAF-12\_Larvae-L3-stage | 15 | 6.17 | 1.8e-06 |
| unc-62 | UNC-62\_Larvae-L2-stage | 22 | 3.98 | 2.2e-06 |
| nhr-2 | NHR-2\_Embryos | 26 | 3.31 | 3.9e-06 |
| nhr-12 | NHR-12\_Larvae-L2-stage | 10 | 10.29 | 3.9e-06 |
| aly-2 | ALY-2\_Larvae-L2-stage | 17 | 4.68 | 9.3e-06 |
| nhr-116 | NHR-116\_Larvae-L2-stage | 11 | 7.02 | 3.4e-05 |
| sax-3 | SAX-3\_Larvae-L3-stage | 25 | 3.03 | 3.5e-05 |
| unc-39 | UNC-39\_Embryos | 16 | 4.13 | 1.0e-04 |
| zip-2 | ZIP-2\_Larvae-L4-stage | 14 | 4.06 | 4.9e-04 |
| mef-2 | MEF-2\_Fed-L1-stage-larvae | 15 | 3.79 | 5.2e-04 |
| unc-39 | UNC-39\_Larvae-L2-stage | 11 | 3.93 | 5.0e-03 |
| nhr-23 | NHR-23\_Larvae-L2-stage | 9 | 4.78 | 5.0e-03 |
| ztf-11 | ZTF-11\_Embryos | 10 | 3.93 | 9.6e-03 |
| ztf-11 | ZTF-11\_Larvae-L3-stage | 7 | 4.66 | 2.7e-02 |
| peb-1 | PEB-1\_Larvae-L2-stage | 12 | 2.87 | 3.3e-02 |
| ama-1 | AMA-1\_Larvae-L3-stage | 19 | 2.15 | 4.0e-02 |
